# Crystal Growth Promotion and Defects Healing Enable Minimum Open‐Circuit Voltage Deficit in Antimony Selenide Solar Cells

**DOI:** 10.1002/advs.202105142

**Published:** 2022-01-28

**Authors:** Guangxing Liang, Mingdong Chen, Muhammad Ishaq, Xinru Li, Rong Tang, Zhuanghao Zheng, Zhenghua Su, Ping Fan, Xianghua Zhang, Shuo Chen

**Affiliations:** ^1^ Shenzhen Key Laboratory of Advanced Thin Films and Applications Key Laboratory of Optoelectronic Devices and Systems of Ministry of Education and Guangdong Province College of Physics and Optoelectronic Engineering Shenzhen University Shenzhen 518060 P. R. China; ^2^ CNRS ISCR (Institut des Sciences Chimiques de Rennes) UMR 6226 Université de Rennes Rennes F‐35000 France

**Keywords:** absorber layer engineering, crystal growth, defects healing, open‐circuit voltage deficit, Sb_2_Se_3_ solar cells

## Abstract

Antimony selenide (Sb_2_Se_3_) is an ideal photovoltaic candidate profiting from its advantageous material characteristics and superior optoelectronic properties, and has gained considerable development in recent years. However, the further device efficiency breakthrough is largely plagued by severe open‐circuit voltage (*V*_OC_) deficit under the existence of multiple defect states and detrimental recombination loss. In this work, an effective absorber layer growth engineering involved with vapor transport deposition and post‐selenization is developed to grow Sb_2_Se_3_ thin films. High‐quality Sb_2_Se_3_ with large compact crystal grains, benign [hk1] growth orientation, stoichiometric chemical composition, and suitable direct bandgap are successfully fulfilled under an optimized post‐selenization scenario. Planar Sb_2_Se_3_ thin‐film solar cells with substrate configuration of Mo/Sb_2_Se_3_/CdS/ITO/Ag are constructed. By contrast, such engineering effort can remarkably mitigate the device *V*_OC_ deficit, owing to the healed detrimental defects, the suppressed interface and space‐charge region recombination, the prolonged carrier lifetime, and the enhanced charge transport. Accordingly, a minimum *V*_OC_ deficit of 0.647 V contributes to a record *V*_OC_ of 0.513 V, a champion device with highly interesting efficiency of 7.40% is also comparable to those state‐of‐the‐art Sb_2_Se_3_ solar cells, paving a bright avenue to broaden its scope of photovoltaic applications.

## Graphical abstract

A two‐step thermodynamic/kinetic deposition process involving vapor transport deposition and post‐selenization is developed to grow high‐quality Sb_2_Se_3_ thin films. Such absorber engineering can heal detrimental defects, prolong carrier lifetime, suppress interface, and space‐charge region recombination. Thus, the substrate structured Mo/Sb_2_Se_3_/CdS/ITO/Ag solar cell delivers a record open‐circuit voltage (*V*_OC_) of 0.513 V with minimum deficit, and highly competitive efficiency of 7.40%.

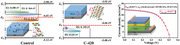

## 1 Introduction

Thin‐film photovoltaic (TFPV) technologies have attracted tremendous research attention thanks to the advantages of low material consumption, high power generation, and scalable flexibility.^[^ ^1^, ^2^, ^3^, ^4^ ^]^ Among various kinds of thin‐film solar cells, remarkable successes have been achieved in the representative cadmium telluride (CdTe), copper indium gallium selenide (CIGS), and perovskites with certified power conversion efficiencies (PCEs) exceeding 22% at laboratory scale.^[^ ^5^ ^]^ However, the scarcity (e.g., In, Ga, and Te), toxicity (e.g., Cd, Pb) of the constituent elements, and/or the natural instability might restrict their large‐scale applications. Thus, some alternative environment‐friendly, cost‐efficient, and intrinsically‐stable TFPV materials are getting growing concerns.^[^ ^6^, ^7^ ^]^ Antimony selenide (Sb_2_Se_3_) has emerged as a highly promising candidate owing to its abundant reserves, low toxicity, superior stability, and excellent optoelectronic properties, including ideal bandgap (*E*_g_ of 1.1–1.3 eV, close to the optimal value for single‐junction solar cell), high absorption coefficient (>10^5^ cm^−1^), decent carrier mobility (≈10 cm^2^ V^−1^ s^−1^) and long carrier lifetime (≈60 ns).^[^ ^8^, ^9^, ^10^, ^11^ ^]^ Furthermore, Sb_2_Se_3_ possesses a 1D crystal structure accumulated by [Sb_4_Se_6_]*_n_* nanoribbons with van der Waals (vdW) forces along the [100] and [010] axes, strong covalent bonds along [001] axis.^[^ ^12^, ^13^ ^]^ Thus, the grain boundaries are intrinsically benign as long as the atomic chains are oriented perpendicular to the substrate, which is also beneficial for strengthening carrier transport and reducing recombination losses.^[^ ^12^ ^]^ A Shockley‐Queisser (S‐Q) theory determined PCE exceeding 30% really demonstrates its great application potential as high efficiency TFPV device.^[^ ^14^ ^]^

In the last decade, Sb_2_Se_3_ solar cells have been extensively investigated, unveiling considerable progress with PCEs of 3.21%,^[^ ^15^ ^]^ 7.6%,^[^ ^9^ ^]^ and 9.2%^[^ ^8^ ^]^ for mesoporous sensitized‐type, planar heterojunction geometry‐type and core–shell structured nanorod array Sb_2_Se_3_ solar cells, respectively. However, it is still far behind its S‐Q limit efficiency. Statistical analysis of key performance parameters shows that the short‐circuit current density (*J*_SC_) and fill factor (FF) of the state‐of‐the‐art Sb_2_Se_3_ solar cells can be optimized to 70% of their S‐Q limits. But the ratio of open‐circuit voltage (*V*_OC_) to its S‐Q limit (*V*_OC_^SQ^, equals to 0.941  × *E*_g_/*q* − 0.171 V) is mostly less than 50%, indicating severe *V*_OC_ deficit (defined as *E*_g_/*q* − *V*_OC_).^[^ ^16^ ^]^ According to the balance principle, the net loss at *V*_OC_ shows a quasi‐linear relationship with *E_g_*, thus a minimum *V*_OC_ deficit is about 0.24 V regarding *E*_g_ of 1.2 eV for Sb_2_Se_3_.^[^ ^17^ ^]^ However, the *V*_OC_ deficit of the highest efficiency Sb_2_Se_3_ solar cell is greater than 0.8 V, much worse than that of perovskite (0.291 V), c‐Si (0.360 V), organic (0.490 V), chalcogenide‐based CIGS (0.346 V), CdTe (0.593 V), and even CZTSSe (0.617 V) solar cells,^[^ ^5^ ^]^ which has become the core bottleneck for further breakthrough of device efficiency. In this perspective, Tang et al. conducted a systematic analysis of *V*_OC_ loss with respect to the intrinsic material properties and device characteristics.^[^ ^16^ ^]^ It claimed that the complicated deep defects (e.g., V_Se_, Sb_Se_, Se_Sb_), short carrier lifetime (0.1–1 ns), low doping density (≈10^13^ cm^−3^), large Urbach energy (30–40 meV), as well as the inevitable interface recombination and space‐charge region (SCR) recombination are the main challenges. Actually, to cope such issues, various strategies have been attempted, such as absorber engineering of post‐selenization,^[^ ^18^ ^]^ co‐evaporation of Sb_2_Se_3_/Se,^[^ ^19^ ^]^ and external doping,^[^ ^20^ ^]^ interfaces engineering of band alignment modification or hole transport layers introduction.^[^ ^21^, ^22^ ^]^

Despite those great efforts, the results are still unsatisfactory. For heterojunction thin‐film solar cells, the absorber material as well as the interface quality synergistically determine the final device efficiency. Thus, high‐quality Sb_2_Se_3_ absorber layer should be first prepared with controllable deposition processes and growth dynamics. Various film deposition techniques, such as hydrothermal deposition,^[^ ^23^ ^]^ solution processing,^[^ ^24^ ^]^ thermal (or rapid thermal) evaporation,^[^ ^12^, ^25^ ^]^ close‐spaced sublimation (CSS),^[^ ^26^ ^]^ pulsed‐laser deposition (PLD),^[^ ^27^ ^]^ magnetron sputtering deposition (MSD),^[^ ^18^ ^]^ and vapor transport deposition (VTD),^[^ ^9^ ^]^ have been developed to prepare Sb_2_Se_3_ absorber layer. Among them, the VTD processed Sb_2_Se_3_ thin film showed an improved crystallinity, reduced deep defects, and suppressed trap‐assisted recombination, leading to a superstrate structured champion Sb_2_Se_3_ solar cell (PCE = 7.6%, *V*_OC_ = 0.42 V), as reported by Tang et al.^[^ ^9^ ^]^ In parallel, our group recently displayed a self‐assembled growth of Sb_2_Se_3_ thin film with large crystal grains, benign preferential orientation, and accurate chemical composition via an effective MSD and post‐selenization involved process, resulting in an interesting substrate structured Sb_2_Se_3_ solar cell (PCE = 6.84%, *V*_OC_ = 0.504 V).^[^ ^21^ ^]^ Thus, combining the advantageous VTD process (e.g., adjustable operating possibility, low cost and fast turnaround) and the compensatory post‐selenization heat atmosphere to control Sb_2_Se_3_ thin film growth kinetics, which might effectively diminish the bulk defects/impurities and/or surface/interface trap states induced *V*_OC_ deficit, and therefore improve the device *V*_OC_ and PCE, that really seems interesting and needs exploration. In addition, the substrate device configuration has some advantages like tailoring the absorber layer independently and engineering the interface efficiently,^[^ ^28^ ^]^ which matches well with this proposed Sb_2_Se_3_ scenario, and is really worth in‐depth investigation.

In this work, an effective absorber layer growth engineering of pre‐VTD combined with post‐selenization was developed to prepare Sb_2_Se_3_ thin films. High‐quality Sb_2_Se_3_ with large compact grains, benign [hk1] growth orientation, and stoichiometric composition were successfully fulfilled under an optimized post‐selenization scenario. Sb_2_Se_3_ thin‐film solar cells with substrate configuration of Mo/Sb_2_Se_3_/CdS/ITO/Ag were constructed. The additional post‐selenization heat treatment has been demonstrated to remarkably improve the device *V*_OC_ and PCE, which can be ascribed to the improved absorber layer and heterojunction quality, the reduced interfacial and bulk defects as well as the defects‐assisted recombination. As a result, a champion device with highly competitive PCE of 7.40% was achieved, a minimum *V*_OC_ deficit of 0.647 V contributed to a record *V*_OC_ of 0.513 V for all reported Sb_2_Se_3_ solar cells, paving a bright avenue to broaden its scope of TFPV applications.

## 2 Results and Discussion

**Figure 1:**
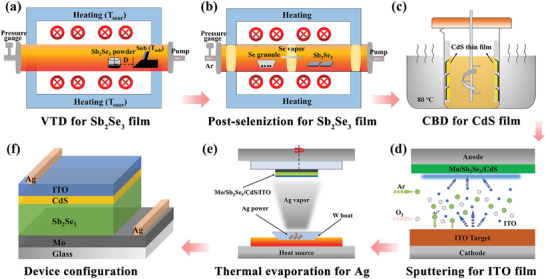
Schematic illustration of the preparation process of the substrate structured Sb_2_Se_3_ thin‐film solar cell. a) VTD process for Sb_2_Se_3_ thin film. b) Post‐selenization heat treatment of Sb_2_Se_3_. c) CdS buffer layer deposited via chemical bath deposition (CBD) method. d) ITO layer deposited by magnetron sputtering. e) Ag electrode prepared via thermal evaporation process. f) Schematic configuration of the final Sb_2_Se_3_ device.

**Figure 2:**
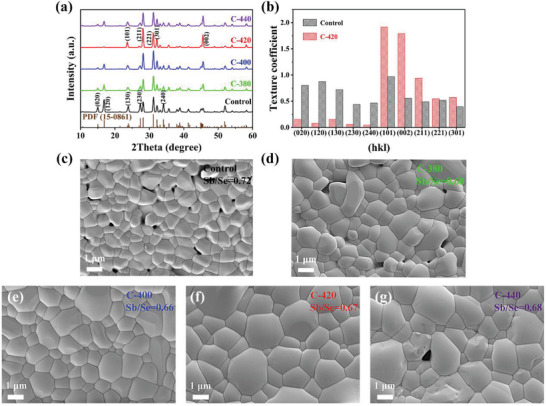
a) XRD patterns of the VTD processed and post‐selenized Sb_2_Se_3_ thin films. b) Texture coefficients of the diffraction peaks of the representative Control and C‐420 Sb_2_Se_3_ thin films. c–g) SEM top‐view images and the corresponding EDS derived Sb/Se ratio of the Sb_2_Se_3_ thin films with different post‐selenization temperature, labeled as Control, C‐380, C‐400, C‐420, and C‐440, respectively.

As shown in **Figure** **1**, a two‐step thermal deposition process involving pre‐VTD and post‐selenization was developed to prepare Sb_2_Se_3_ thin films. **Figure** **2**a shows the XRD patterns of the as‐prepared Sb_2_Se_3_ thin films, including the VTD processed pristine sample (Control), and the counterparts underwent post‐selenization at different temperatures (i.e., 380, 400, 420, and 440°C), which are denoted as C‐380, C‐400, C‐420, and C‐440, respectively. All the samples exhibit prominent peaks in good agreement with the JCPDS standard card (No. 15–0861) of the orthorhombic phase of Sb_2_Se_3_ without a second phase, indicating the absence of any detectable impurity. An observable increase of diffraction peak intensities under appropriate post‐selenization heat treatment, e.g., at temperatures of 400 and 420°C, suggesting thermally induced crystal re‐growth and crystallinity improvement of the Sb_2_Se_3_ thin films under Se atmosphere. Notably, the diffraction peaks of (120), (130), (230), and (240) show sharp characteristics, indicating a preferential growth orientation of [hk0] for Control sample. However, an obvious evolution to the dominant [hk1] growth orientation can be observed for the post‐selenized samples, especially for C‐420 thin film. In order to evaluate it more intuitively, the texture coefficient (TC) of the major diffraction peaks were calculated based on the following equation^[^ ^29^ ^]^

(1) $$TC_{\mathrm{hkl}}=\frac{I_{\left(\mathrm{hkl}\right)}}{I_{0\left(\mathrm{hkl}\right)}}\;/\left(\frac{1}{N}\sum_{i\;=\;1}^{N}\frac{I_{\left(h_{i}k_{i}l_{i}\right)}}{I_{0\left(h_{i}k_{i}l_{i}\right)}}\right)$$

where *I*_(hkl)_ and *I*_0(hkl)_ are the diffraction peak intensities of [hkl] planes in the measured and standard XRD pattern of Sb_2_Se_3_, respectively. Large TC value of a diffraction peak indicates preferred orientation along this particular direction. The significant variation of TC values associated to Control and C‐420 thin films are shown in Figure 2b, driving the further investigation of growth mechanism under this VTD and post‐selenization preparation scenario. The VTD processed Sb_2_Se_3_ thin film is substantially related to a substrate‐film interaction, the Mo substrate has been reported to preferentially induce the formation of [hk0] nuclei, leading to the slow growth rate and [hk0] oriented films.^[^ ^30^ ^]^ However, during post‐selenization heat treatment, the energetic ambient Se vapor could have a considerable effect on adatoms behavior and nuclei‐substrate interaction, and therefore alter growth kinetics of the thin films with specific morphology and orientation. The top‐view SEM images and the atomic Sb/Se ratios of the Sb_2_Se_3_ thin films are also shown in Figure 2c–g. For Control sample, Sb_2_Se_3_ thin film with small grains and poor compactness can be observed, along with distinct micro‐voids on the surface. SEM‐coupled EDS result also revealed that the VTD processed thin film was severely Se poor with a deviated Sb/Se atomic ratio of 0.72, which can be attributed to the weight loss of Sb_2_Se_3_ at a threshold temperature (≈423°C), and the high vapor pressures of Sb_2_Se_3_ and Se.^[^ ^31^ ^]^ After post‐selenization heat treatment, the Sb_2_Se_3_ thin films exhibited a gradual increase of compactness and uniformity with increasing the temperature from 380 to 420°C, accompanied by a synchronous increase of average grain size from ≈0.8 µm (Control) to 1.6 µm (C‐420). The detailed frequency histograms versus grain size distribution are depicted in Figure S1 (Supporting Information). Moreover, the severe Se deficit can be mitigated upon post‐selenization, until achieving an optimal Sb/Se ratio of 0.67 that close to the standard stoichiometric ratio value for Sb_2_Se_3_. The post‐selenization duration‐dependent crystallinity, morphologies, and chemical compositions also showed a similar positive correlation evolution (Figure S2, Supporting Information). Accordingly, a growth model involved with re‐crystallization and re‐growth of the Control Sb_2_Se_3_ thin film upon post‐selenization heat treatment can be proposed, which will be clarified later. Finally, once an excessively high post‐selenization temperature (e.g., 440°C) was applied, the diffraction peak intensities would decrease, the detrimental micro‐voids would emerge again, and the chemical composition would deviate reversely, which was possibly caused by a partial decomposition under its high vapor pressure nature (Figure 2a,g).

**Figure 3:**
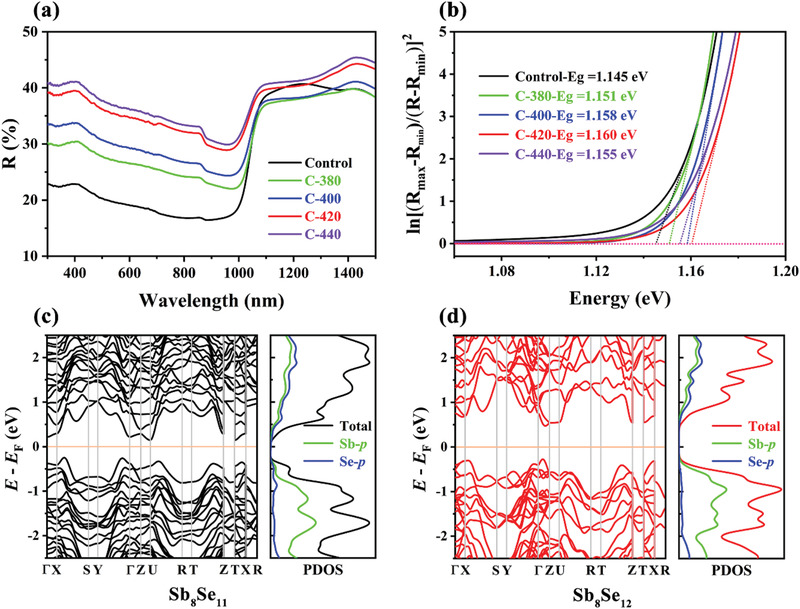
Optical characterizations of the Sb_2_Se_3_ thin films: a) Reflection spectra, b) Plots of ln[(*R*_max_ − *R*_min_)/(*R* − *R*_min_)]^2^ versus Energy, used to obtain direct bandgap. DFT calculations determined electronic structure and orbital DOS information of the representative c) Sb_8_Se_11_ and d) Sb_8_Se_12_ models.

The investigation of optical properties of the as‐prepared thin films is essential for understanding and/or engineering its high efficiency TFPV devices. The reflection spectra of the Sb_2_Se_3_ thin films were first obtained via UV/Vis/NIR spectrophotometer on glass substrate, and covering a wavelength range of 300–1500 nm (**Figure** **3**a). Accordingly, the reflectance of the Control thin film was lower than that of the post‐selenized thin films in the short wavelength absorption region, and also revealed a gradual increase of the values with increasing the post‐selenization temperature. It was closely related to the crystallinity‐dependent refractive index, the grain size and surface compactness evolution, as corroborated by the XRD and SEM results. Moreover, a synchronous blue‐shift of the absorption cut‐off edge could be observed, implying the variation of band structure. Thus, the important indicator of optical bandgap (*E*_g_) was specifically calculated according to the following formulas

(2) $$2\alpha d\;=\ln\left[\left(R_{\max}-R_{\min}\right)/\left(R-R_{\min}\right)\right]\;$$

(3) $$\alpha hv\;=C{\left(hv-E_{g}\right)}^{n}\;$$

where *α* is the absorption coefficient, d is thickness, the reflectance falls from *R*_max_ to *R*_min_ due to the intrinsic absorption of light by the material.^[^ ^32^ ^]^ The latter is a typical *T*_auc_ formula, where *C* is a constant, *h* is the Planck's constant, *v* is the photon frequency, *n* is an index.^[^ ^33^ ^]^ The calculated *E*_g_ values were 1.145, 1.151, 1.158, 1.160, and 1.155 eV for Control, C‐380, C‐400, C‐420, and C‐440 thin films, respectively. Such *E*_g_ value was close to the optimal value for efficient solar light harvesting, an observable variation also matched well with the real chemical composition, i.e., Sb‐rich (Se‐poor) or Se‐rich (Se‐compensation) conditions. To further verify this composition‐dependent band structure evolution, DFT calculations with exchange‐correlation functional under the generalized gradient approximation (GGA) were performed on the representative Sb_8_Se_11_ (corresponds to Control sample) and Sb_8_Se_12_ (corresponds to C‐420 sample) models (Figure S3 and Note S1, Supporting Information).^[^ ^34^ ^]^ As shown in Figure 3c,d, by contrast, the latter structure presents an enlarged *E*_g_ value, accompanied by more significant direct bandgap characteristic, which is consistent with the experimental observations. The corresponding orbital‐resolved projected density of state (DOS) plots further suggest that the VBM is dominated by Se‐p orbitals, whereas the CBM consists of Se‐p and Sb‐p orbitals. Thus, for Sb‐rich Control sample, once the Se is replaced by Sb, the new incorporated Sb will bond with the surrounding Sb to induce p–p hybridization, the bonding state will decrease its *E*_g_. Moreover, the DFT calculations also shows that the conductivity type can be tuned form n‐type to p‐type through tuning the Se chemical potential from Sb‐rich to Se‐rich condition. According to the literature, it is reasonable to occur since the dominant defects change from donor defects to acceptor defects.^[^ ^35^, ^36^ ^]^ Overall, this Sb_2_Se_3_ growth engineering can provide opportunity to modify its electronic structures and optical properties, making it an attractive candidate for TFPV applications.

**Figure 4:**
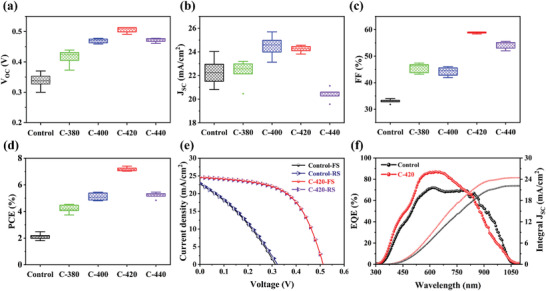
Statistical distribution of the key performance parameters of the Sb_2_Se_3_ thin‐film solar cells, including a) *V*_OC_, b) *J*_SC_, c) FF, and d) PCE. e) *J–V* curves of the representative Control and C‐420 devices under forward scan (FS) and reverse scan (RS). f) EQE and integrated *J*_SC_ of the devices.

The controllable Sb_2_Se_3_ thin films were used to construct substrate structured solar cells with configuration of Mo/Sb_2_Se_3_/CdS/ITO/Ag, and the statistical distributions of the key performance parameters are presented in **Figure** **4**a–d. All the five series of devices, each with a set of twenty synchronously fabricated sub‐cells demonstrated satisfactory reproducibility. By contrast, the post‐selenized devices possessed an obvious increase of open‐circuit voltage (*V*_OC_) and fill factor (FF), as well as a slight increase of short‐circuit current density (*J*_SC_) under appropriate treatment of Sb_2_Se_3_ thin films. Therefore, PCE as a product of *V*_OC_ × *J*_SC_ × FF can be improved via this important absorber layer engineering. The detailed champion device performance parameters of each category are summarized in **Table** **1**. *J–V* curves of the representative Control and C‐420 devices are shown in Figure 4e. Accordingly, under the simulated AM 1.5G solar irradiation, the Control device offered a *J*_SC_ of 22.72 mA cm^−2^, an inferior *V*_OC_ of 312 mV, and FF of 34.02%, thus resulting in an unsatisfactory PCE of 2.41%. In comparison, an obvious improvement of PCE to 7.40% was observed for C‐420 device, presenting simultaneously increased *J*_SC_, *V*_OC_, and FF to 24.56 mA cm^−2^, 513 mV, and 58.74%, respectively. Such great breakthrough matched well with the high‐quality C‐420 thin film with higher crystallinity, better orientation, larger and void‐free grain characteristics, which could effectively reduce bulk and/or interfacial defects, suppress transport and/or recombination loss. Moreover, a further comparison with some previously reported state‐of‐the‐art Sb_2_Se_3_ solar cells prepared via VTD or post‐selenization involved MSD techniques is shown in Figure S4 (Supporting Information) and **Table** **2**.^[^ ^9^, ^18^, ^21^, ^37^ ^]^ It is worth mention that this interesting efficiency of 7.40% is higher than the MSD processed counterpart (highest PCE of 6.84%),^[^ ^20^ ^]^ comparable to the VTD processed superstrate structured Sb_2_Se_3_ solar cells (champion PCE of 7.6%, also the record value for planar‐type Sb_2_Se_3_ solar cells),^[^ ^9^ ^]^ and is much superior than the VTD processed substrate structured device (PCE of 5.35%).^[^ ^37^ ^]^ Furthermore, a maximum *V*_OC_ of 513 mV undoubtedly represents the highest value for all Sb_2_Se_3_ solar cells reported so far. Regarding the aforementioned biggest challenge of *V*_OC_ deficit, herein, the corresponding values are also calculated and summarized in Table 2. Interestingly, the *V*_OC, def_ of the champion C‐420 device can be reduced to 0.647 V, which is much lower than that of other efficient Sb_2_Se_3_ solar cells reported in literature, confirming the effectiveness of this VTD and post‐selenization coupled absorber layer growth engineering in breaking through the core bottleneck. As shown in Figure 4e, the forward and reversed biased scanned *J–V* curves of the champion C‐420 device could perfectly overlap, reflecting no observable hysteresis loss under its high‐quality Sb_2_Se_3_ absorber layer, superior heterojunction contact, and benign device interface. Moreover, the champion C‐420 device presented an outstanding long‐term stability since only a slight performance degradation could be seen with PCE decreased from initial 7.40% to the final 7.31% after 60 days storage in ambient air without encapsulation (Figure S5, Supporting Information). EQE spectra and the corresponding integrated *J*_SC_ of the Control and C‐420 devices are portrayed in Figure 4f. Both devices exhibited broad photo response range from UV to NIR, echoing the intrinsically narrow bandgap of Sb_2_Se_3_ with strong light absorption. The higher EQE values for the C‐420 device in the wavelength range of 300–800 nm can be attributed to the reduced non‐radiative recombination losses, i.e., the interface recombination and SCR recombination.^[^ ^38^ ^]^ A slightly higher photocurrent generation for the Control device in the wavelength greater than 850 nm was possibly due to the absorption of more infrared photons under a smaller bandgap and less reflection loss. The integrated *J*_SC_ values calculated from EQE data were 22.15 and 24.45 mA cm^−2^ for Control and C‐420 devices, respectively, which were close to the corresponding *J*_SC_ values obtained from *J–V* measurement results.

**Table 1:** Device performance parameters based on Sb_2_Se_3_ absorber layer with different post‐selenization conditions

Devices	*V*_OC_ [V]	*J*_SC_ [mA cm^−2^]	FF [%]	PCE [%]
Control	0.312	22.72	34.02	2.41
C‐380	0.424	22.57	47.41	4.53
C‐400	0.478	25.69	44.33	5.44
C‐420	0.513	24.56	58.74	7.40
C‐440	0.478	20.26	55.54	5.47

**Table 2:** A comparison of photovoltaic parameters of Sb_2_Se_3_ devices prepared via different methods

Method	Device configuration	PCE [%]	*V*_OC_ [V]	*J*_SC_ [mA cm^−2^]	FF [%]	*V*_OC_, _def_ [V] *E*_g_ */q*−*V*_OC_	Ref.
VTD^a)^	ITO/CdS/Sb_2_Se_3_/Au	7.60	0.420	29.90	60.40	0.770	Tang^[^ ^9^ ^]^
VTD^a)^	Mo/Sb_2_Se_3_/In_2_S_3_/ZnO/ITO	5.35	0.370	28.22	51.90	0.820	Tang^[^ ^37^ ^]^
MSD‐Se^b)^	Mo/Sb_2_Se_3_/CdS/ITO/Ag	6.06	0.494	25.91	47.70	0.716	Liang^[^ ^18^ ^]^
MSD‐Sb‐Se^c)^	Mo/Sb_2_Se_3_/CdS/ITO/Ag	6.84	0.504	24.91	54.47	0.651	Liang^[^ ^21^ ^]^
VTD^a)^	Mo/Sb_2_Se_3_/CdS/ITO/Ag	2.41	0.312	22.72	34.02	0.833	This work
VTD‐Se^d)^	Mo/Sb_2_Se_3_/CdS/ITO/Ag	7.40	0.513	24.56	58.74	0.647	This work

^a)^ Vapor transport deposition

^b)^ Sputtering Sb_2_Se_3_ and post‐selenization

^c)^ Sputtering Sb precursor and post‐selenization

^d)^ Vapor transport deposition Sb_2_Se_3_ and post‐selenization.

After comparing the device parameters, the mechanism of a high *V*_OC_ (i.e., minimum *V*_OC_ deficit) in this work should be emphatically elucidated. The *V*_OC_ is strongly dependent on the diode ideality factor (*A*) and reverse saturation current (*J*_0_) according to Equation (4)^[^ ^39^ ^]^

(4) $$V_{\mathrm{OC}}=\frac{AkT}{q}\;\ln\left(\frac{J_{\mathrm{SC}}}{J_{0}}+1\right)$$

The values of *A*, *J*_0_, along with the shunt conductance (*G*) and resistance (*R*) were calculated by treating the dark *J–V* characteristic curves. To be illustrative, the current signals of the Control device would fluctuate severely during the measurement in dark under applied bias voltage of −1 to 1 V (Figure S6, Supporting Information). Thus, dark *J–V* curves of the C‐400 and C‐420 devices with obvious rectifying characteristics were compared and explored according to a single exponential diode equation^[^ ^40^ ^]^

(5) $$J\;=J_{0}\exp\left[\frac{q}{AkT}\;\left(V-JR\right)\right]+GV-J_{L}$$

**Figure 5:**
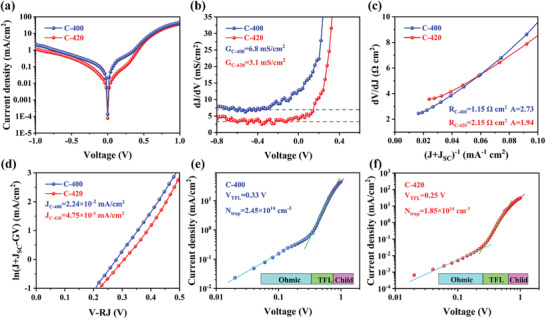
Electrical behaviors of the representative C‐400 and C‐420 devices: a) Dark *J–V* curves, b) shunt conductance *G* characterizations, c) series resistance *R* and ideality factor *A* characterizations, d) reverse saturation current density *J*_0_ characterizations. e,f) Logarithmic *J–V* curves of the C‐400 and C‐420 devices, respectively, showing Ohmic, TFL and Child region.

The values of *G* were first extracted from the flat portions under reverse bias of plot of *dJ/dV* against *V* (**Figure** **5**b), and the obtained values were 3.1 and 6.8 mS cm^−2^ for C‐400 and C‐420 devices, respectively. Figure 5c shows the plots of *dV/dJ* in relation to (*J+J*_SC_) ^−1^, then the series resistance *R* and diode ideality factor *A* could be obtained through the intercept of y‐axis and a slop of *AkT/q*, respectively. The *R* values were linear fitted as 1.15 and 2.15 Ω cm^2^ for C‐400 and C‐420, respectively. The obtained values of A were 2.73 and 1.94, implying the coexistence of interface and SCR recombination in both two devices, whereas an obvious decrease of SCR recombination induced by the trap levels in the depletion region can be observed for C‐420. Finally, the reverse saturation current *J*_0_ were extracted from the plot of ln(*J+J*_SC_−*GV*) against *V*−*RJ* (Figure 5d), the corresponding intercept yielded an *J*_0_ of 2.24 × 10^−2 ^mA cm^−2^ for C‐400, and 4.75 × 10^−3 ^mA cm^−2^ for C‐420. Thus, under a more appropriate post‐selenization heat treatment of Sb_2_Se_3_ absorber layer, a lower reverse saturation current and better diode ideality factor established a much superior Sb_2_Se_3_/CdS junction quality, justifying higher *V*_OC_ and PCE for C‐420 device. Moreover, these critical electrical parameters were also close to that of highly efficient (e.g., PCE of 7.49%) VTD processed Sb_2_Se_3_ solar cell in superstrate configuration.^[^ ^41^ ^]^ In order to further understand the defect states of these engineered Sb_2_Se_3_ thin films, the standard space charge limited current (SCLC) model was applied. Figure 5e,f shows the logarithmic *J–V* curves of the C‐400 and C‐420 devices, respectively. The curves can be divided into three regimes: the ohmic region (at low voltages, exponent n = 1), the trap‐filled limit (TFL) region (at intermediate voltages, *n* > 3), and the trap‐free Child region (at high voltages, *n* > 2). In the TFL region, the current snappishly upsurges once the bias voltage exceeds the kink point, signifying that the trap‐states are completely filled by the injected carriers. Thus, the determined onset voltages (*V*_TFL_) associated to C‐400 and C‐420 were 0.33 and 0.25 V, respectively. Then the trap state density *N*_trap_ can be calculated according to the following equation^[^ ^42^ ^]^

(6) $$N_{\mathrm{trap}}=\frac{2\varepsilon_{0}\varepsilon_{r}V_{\mathrm{TFL}}}{qL^{2}}\;$$

where *L* is the thickness of Sb_2_Se_3_ thin film, *q* is the elementary charge, *ε*_0_ is the vacuum permittivity, and *ε*_r_ is the relative permittivity (i.e., 15.1 for Sb_2_Se_3_). The obtained *N*_trap_ values fallen from 2.45 × 10^14^ for C‐400 to 1.85 × 10^14^ cm^−3^ for C‐420, suggesting the latter post‐selenized Sb_2_Se_3_ thin film possessed less trap sites and defect centers. It also agreed well with its more superior quality with larger grains, benign orientation and accurate composition.

It is known that interface recombination and SCR recombination are two kinds of common recombination mechanisms in TFPV devices, and the dominated recombination loss resulted in severe *V*_OC_ deficit. Herein, the recombination mechanism referred to where and how the recombination occurred was systematically investigated. First, temperature‐dependent open‐circuit voltage (*V*_OC_‐*T*) measurement was carried out to obtain the activation energy (*E*_a_), according to the following equation^[^ ^43^ ^]^

(7) $$E_{a}=\;qV_{\mathrm{OC}}+\;AkT/\ln\left(J_{0}/J_{L}\right)$$

**Figure 6:**
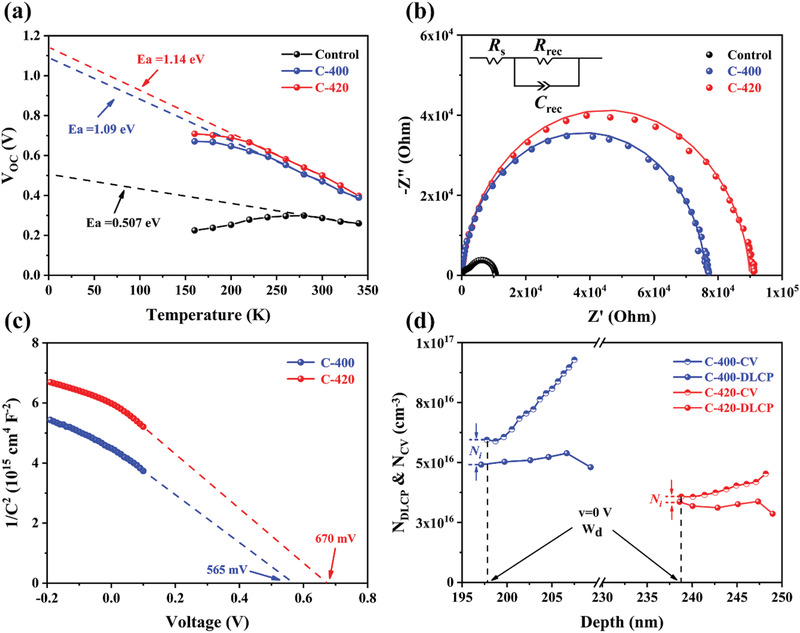
a) Temperature‐dependent *V*_OC_ measurements, and b) Nyquist plots of the representative Control, C‐400 and C‐420 Sb_2_Se_3_ devices. c) 1/*C^2^‐V* plots, and d) *C–V* and *DLCP* profiles of the C‐400 and C‐420 devices.

Hence, *E*_a_ value could be calculated by extrapolating the *V*_OC_ to *y*‐axis (*T* = 0 K). In principle, the *E*_a_ of interface recombination is lower than *E*_g_, and *E*_a_ of SCR recombination is equal to *E*_g_.^[^ ^10^ ^]^ As shown in **Figure** **6**a, *E*_a_ value of the Control device was 0.51 eV, much lower than its *E*_g_ value (1.145 eV), suggesting a dominated defect‐assisted interface recombination, which might be originated from the poor interfacial adhesion between Sb_2_Se_3_ absorber layer and other functional layers due to the existence of multiple micro‐voids. Both C‐400 and C‐420 devices possessed an enlarged *E*_a_ value, especially an *E*_a_ of 1.14 eV that was quite close to its *E*_g_ of 1.160 eV (C‐420), authenticating suppressed overall recombination loss with mitigatory SCR recombination in dominant. Electrochemical impedance spectroscopy (EIS) analysis was also conducted to examine the charge recombination and charge transfer characteristics of the solar cells. A comparison of the Nyquist plots of the representative Control, C‐400, and C‐420 devices are shown in Figure 6b, along with an inset equivalent circuit diagram, where *R*_s_, *R*_rec_ and *C*_rec_ represent series resistance, recombination resistance and chemical capacitance, respectively. Accordingly, the intercept on the *x*‐axis (*Z*′) was assigned to *R*_s_, the extracted *R*_s_ values for Control, C‐400 and C‐420 were 2.85, 1.92, and 1.79 Ω cm^2^, respectively, strengthening the post‐selenized Sb_2_Se_3_ thin films with large grains and benign orientation that would benefit the charge transport. The arc's diameter length was directly corresponding to the *R*_rec_, an obvious suppressed probability of charge recombination at the interface could be observed for C‐420 device, which was also consistent with the *E*_a_ analysis. The *1/C^2^‐V* curves derived from the capacitance‐voltage (*C*–*V*) measurements were plotted to scrutinize the junction quality. Figure 6c shows the corresponding curves of the representative C‐400 and C‐420 devices, the results of Control device were eliminated under its unstable measurement. The heterojunction‐dependent built‐in voltage (*V*_bi_) could be extracted through linear fitting and extrapolating to *x*‐axis. *V*_bi_ of the C‐420 device (670 mV) was larger than that of the C‐400 device (565 mV), indicating an improved Sb_2_Se_3_/CdS heterojunction quality for C‐420. Notably, such an interesting *V*_bi_ belonging to our champion device was higher than that of mostly reported efficient Sb_2_Se_3_ devices, contributing to a record *V*_OC_ in this work.^[^ ^14^, ^18^ ^]^ Drive‐level capacitance profiling (DLCP) characterizations were also performed on C‐420 and C‐440 devices to examine the interfacial defects. In principle, the *C*–*V* determined doping density (*N*_C‐V_) symbolizes the responses from free carriers, bulk defects and interfacial defects, whereas the DLCP measured doping density (*N*_DLCP_) only reveals the responses from free carriers and bulk defects.^[^ ^9^ ^]^ Therefore, the interface defect density (*N*_i_) can be calculated by the discrepancy between *C*–*V* and DLCP profiling at zero bias. The plots of *N*_C‐V_ and *N*_DLCP_ against the profiling depth *x* can be expressed according to the following equations^[^ ^21^ ^]^

(8) $$N_{C-V}=\frac{-2\varepsilon_{r,n}N_{D}}{\left(\frac{d\left(\left(1/C^{2}\right)\right)}{dV}\right)qA^{2}\varepsilon_{0}\varepsilon_{r,n}\varepsilon_{r,p}N_{D}+2\varepsilon_{r,p}}$$

(9) $$N_{\mathrm{DLCP}}=\;-\frac{C_{0}^{3}}{2q\varepsilon_{0}\varepsilon_{r,p}A^{2}C_{1}}$$

(10) $$x\;=\frac{\varepsilon_{0}\varepsilon_{r,p}A}{C_{0}}$$

where *N*_D_ is the doping density of CdS, *A* is the device area, *ε*_0_, *ε*_r,n_ and *ε*_r,p_ represents the permittivity of free space, the relative permittivity of CdS and Sb_2_Se_3_, respectively, *C*_0_ and *C*_1_ are two quadratic fitting parameters derived from the *C*–*V* curves. As shown in Figure 6d, the difference between *N*_C‐V_ and *N*_DLCP_ of the C‐400 device was much larger than that of the C‐420 device, the estimated *N*_i_ values for these two devices were 1.04 × 10^16^ cm^−3^ and 2.24 × 10^15^ cm^−3^ (at *v* = 0 V), respectively. Such significant *N*_i_ decrease with nearly one order of magnitude indicated an improved Sb_2_Se_3_/CdS interface, e.g., smooth contact, benign band alignment, alleviated lattice mismatch, and passivated interface defects for C‐420 device. Furthermore, the C‐420 device possessed a large depletion width (*W*_d_) as compared to C‐400 device, which was reasonable to occur since it varied positively with *V*_bi_ according to the following equation^[^ ^44^ ^]^

(11) $$W_{d}=\sqrt{\frac{2\varepsilon_{p}\varepsilon_{n}{\left(N_{A}+N_{D}\right)}^{2}}{qN_{A}N_{D}\left(\varepsilon_{p}N_{A}+\varepsilon_{n}N_{D}\right)}V_{\mathrm{bi}}}\;$$

where *q* is the elementary charge, *ε*_p_ and *ε*_n_ are the permittivity, *N*_A_ and *N*_D_ are the acceptor density and donor density in Sb_2_Se_3_ and CdS, respectively. Since the doping concentration of CdS is much higher than that of Sb_2_Se_3_, almost the entire *W*_d_ is located within Sb_2_Se_3_ absorber layer. An optimal *W*_d_ of 239 nm for C‐420 is really beneficial for the light absorption and carrier extraction, corresponding to its much superior device performance. All these heterojunction or interface associated parameters are summarized in **Table** **3**. Notably, an efficient Sb_2_Se_3_ absorber layer growth engineering would also influence the junction quality, the interface quality, the recombination mechanism, and therefore improve device *V*_OC_ and PCE.

**Table 3:** Summary of heterojunction and interface associated photovoltaic parameters of the Sb_2_Se_3_ devices

Devices	*J*_0_ [mA cm^−2^]	*A*	*V*_TFL_ [V]	*N*_trap_ [cm^−3^]	*E*_a_ [eV]	*R*_s_ [Ω cm^2^]	*V*_bi_ [V]	*N*_i_ [cm^−3^]
Control	–	–	–	–	0.507	2.85	–	–
C‐400	2.24 × 10^−2^	2.73	0.33	2.45 × 10^14^	1.09	1.92	0.565	1.04 × 10^16^
C‐420	4.75 × 10^−3^	1.94	0.25	1.85 × 10^14^	1.14	1.79	0.670	2.24 × 10^15^

**Figure 7:**
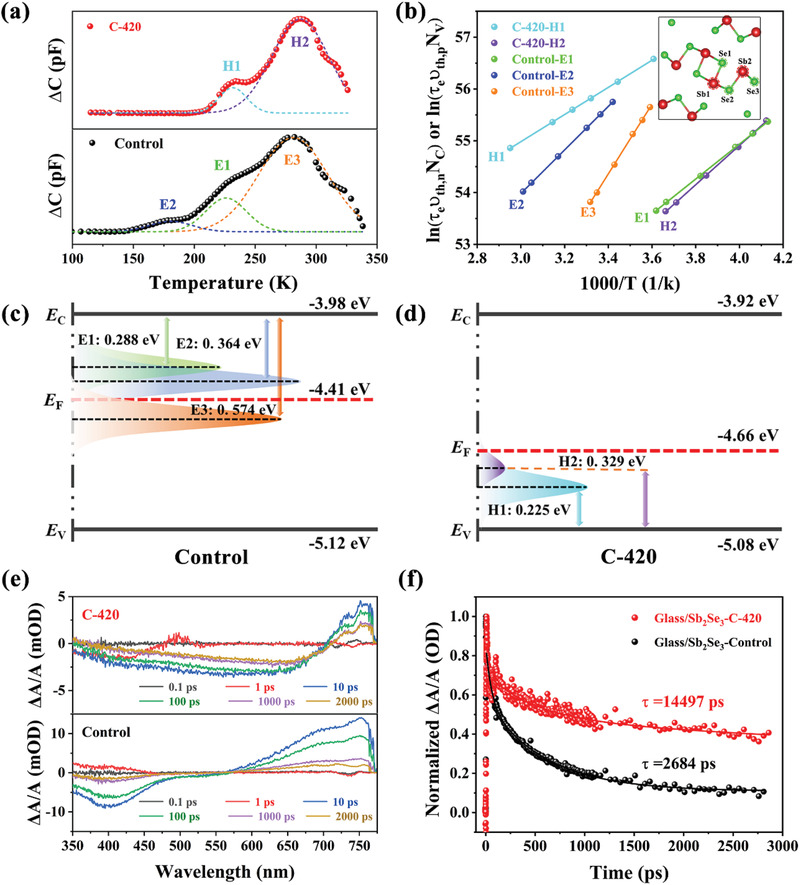
a) DLTS signals from the Control and C‐420 Sb_2_Se_3_ devices, b) The corresponding Arrhenius plots obtained from DLTS signals. c,d) Conduction (*E*_C_) and valence (*E*_V_) band edges, Fermi level (*E*_F_) and defect energy levels of the Control and C‐420 samples, respectively. e) Time‐resolved absorption spectra obtained at various time delays after photoexcitation for the Control and C‐420 Sb_2_Se_3_ thin films. f) Transient kinetic traces showing the decay of the PIA peak at 750 nm for both thin films.

Understanding and controlling the bulk defect properties, especially deep‐level defects, is also essential for photovoltaic devices to reduce detrimental nonradiative SCR recombination and improve device performance, in particular *V*_OC_, which is primarily affected by defect depth, defect density and carrier lifetime. Herein, a well‐established deep‐level transient spectroscopy (DLTS) was carried out to investigate defect dynamics of the Control and C‐420 devices, respectively. According to literature, the positive and negative peaks can be ascribed to majority‐carrier and minority‐carrier traps, respectively.^[^ ^36^ ^]^ Thus, the energy band information of the light absorbing thin film was first examined by ultraviolet photoelectron spectroscopy (UPS), wherein, the Control and C‐420 thin films were determined to be n‐type and p‐type conductive characteristics, respectively (Figure S7 and Note S2, Supporting Information), agreeing with DFT calculation results. Consequently, as shown in **Figure** **7**a, three electron traps that denoted as E1, E2, and E3 can be well identified in the Control device. By contrast, only two hole traps, which are denoted as H1 and H2, can be observed in the C‐420 device. The activation energy *E*_A_ (*E*_T_−*E*_V_ or *E*_C_−*E*_T_, where *E*_T_, *E*_C_ and *E*_V_ represent the defect energy level, conduction and valance bands edge, respectively) of the defects in these two devices were calculated from the Arrhenius plots by fitting the points near the DLTS peaks (Figure 7b and Note S3 in Supporting Information). The defect characteristics including defect type, defect energy level (*E*_T_), defect density (*N*_T_), and capture cross‐section (*σ*) are summarized in **Table** **4**. According to the first‐principle theoretical basis and the reported DLTS experimental results in literature,^[^ ^16^, ^45^ ^]^ considering the defects formation energy and defects energy level, regarding the nonequivalent Sb and Se sites (inset in Figure 7b), E1, E2, and E3 can be readily assigned to selenium vacancies (V_Se2_ and V_Se3_), antisite defects of Sb_Se2_, respectively. They were reasonable to exist in Sb‐rich (Se‐poor) Sb_2_Se_3_ light absorbing film, the higher concentration (≈10^14^ cm^−3^) of Sb_Se_ antisite defects were also consistent with their lower formation energy. After an optimal post‐selenization heat treatment of the Sb_2_Se_3_ thin film, the additional Se atoms would preferentially fill the Se vacancies (i.e., Se‐compensation), and also increase the formation energy of Sb_Se_ defects, resulting in the vanish of those donor defects. The emergence of Sb vacancies (V_Sb_) in some particular region was caused by the decrease of Sb content, some excessive Se was likely to occupy these Sb vacancies to form Se_Sb_ antisites for maintaining the structure stability. Thus, in C‐420 device, two observable hole traps of H1 and H2 with *N*_T_ of 6.09 × 10^13^ and 1.46 × 10^13^ cm^−3^ can be assigned to Se_Sb1_ and V_Sb1_, respectively. Consequently, the Sb_2_Se_3_ absorber engineering could induce an obvious healing effect in defect type and defect concentration, which was highly beneficial for reducing the charge recombination. Figure 7c,d illustrates the band edge positions and defect levels for both devices. For Control device, E1, E2, and E3 electron defects are located at about 0.29, 0.37, and 0.57 eV below *E*_C_, which are close to its intrinsic *E*_F_ position, implying strong pinning effect, resulting in severe trap‐assisted recombination and *V*_OC_ loss. Especially, E3 defect showed a largest *N*_T_ × *σ* value (1.95 cm^−1^), which might act as a major recombination center to diminish carrier lifetime (*τ*_trap_), according to the following equation associated with trap‐assisted Shockley‐Read‐Hall (SRH) recombination^[^ ^16^, ^36^ ^]^

(12) $$\tau_{\mathrm{trap}}=\frac{1}{v_{\mathrm{th}}\sigma N_{T}}\;$$

where *v*_th_ is the carrier thermal velocity, capture cross‐section (*σ*) and defect density (*N*_T_) are obtained from DLTS. By contrast, for C‐420 device, H1 and H2 hole defects are located at 0.23 and 0.33 eV above *E*_V_, which can efficiently alleviate the *E*_F_ pinning effect, and boost the split of the quasi‐Fermi levels of electrons and holes, leading to an enlarged *V*_OC_ for the device. In addition, it is worth noting that the *N*_T_ × *σ* of H1 and H2 are markedly decreased with several orders of magnitude, indicating the holes will be emitted again in a very short time even they are captured by these shallow trap states. Moreover, the value is comparable to those of high efficiency perovskite or CZTSSe solar cells, strengthening our effective effort in defects healing.^[^ ^46^, ^47^ ^]^

**Table 4:** Defect parameters of the representative Control and C‐420 Sb_2_Se_3_ solar cells

Devices	Defects	*E*_T_ [eV]	Type	*σ* [cm^2^]	*N*_T_ [cm^−3^]	*N*_T_ × *σ* [cm^−1^]
Control	E1	*E*_C_−0.288	V_Se2_	3.13 × 10^−19^	8.17 × 10^13^	2.56 × 10^−5^
	E2	*E*_C_−0.367	V_Se3_	1.16 × 10^−18^	1.26 × 10^14^	1.46 × 10^−4^
	E3	*E*_C_−0.574	Sb_Se2_	1.68 × 10^−14^	1.16 × 10^14^	1.95
C‐420	H1	*E*_V_ +0.225	Se_Sb1_	3.35 × 10^−21^	6.09 × 10^13^	2.04 × 10^−7^
	H2	*E*_V_ +0.329	V_Sb1_	5.97 × 10^−18^	1.46 × 10^13^	8.72 × 10^−5^

To further study the carrier transport dynamics, the transient absorption spectroscopy (TAS) characterizations were performed on the VTD processed (Control) and post‐selenized (C‐420) Sb_2_Se_3_ thin films that were deposited on soda‐lime glass. Under the absence of any electron or hole extraction layer, it can directly reflect the relaxation of the exciton to ground state through charge recombination. As shown in Figure 7e, an obvious photo‐induced absorption (PIA) peak located at 750 nm can be observed for both two samples, which can be assigned to the trapping of photo‐generated minority carriers. Subsequently, the kinetic decay curves were fitted via a biexponential model (Figure 7f), the detailed fitting parameters are summarized in Table S1 (Supporting Information). To be specific, the short decay lifetime (*τ*_1_) and long decay lifetime (*τ*_2_) can be attributed to the interface/surface recombination and the bulk defect recombination, respectively.^[^ ^16^ ^]^ Compared to Control sample, a significant increase of both *τ*_1_ and *τ*_2_ for C‐420 Sb_2_Se_3_ thin film indicated that both interface/surface recombination and bulk recombination have been suppressed, especially a dominant bulk recombination mitigation mechanism, echoing the aforementioned DLTS results of defects healing. Moreover, it is worth mentioning that the obtained lifetime (*τ* = 14 497 ps) also represents one of the top values among all antimony chalcogenides (Table S2, Supporting Information). Overall, benefitting from this efficient Sb_2_Se_3_ absorber layer growth engineering, the improved junction quality, the healed deep defects, and the prolonged carrier lifetime could undoubtedly improve the *V*_OC_ and PCE of the Sb_2_Se_3_ thin‐film solar cell.

**Figure 8:**
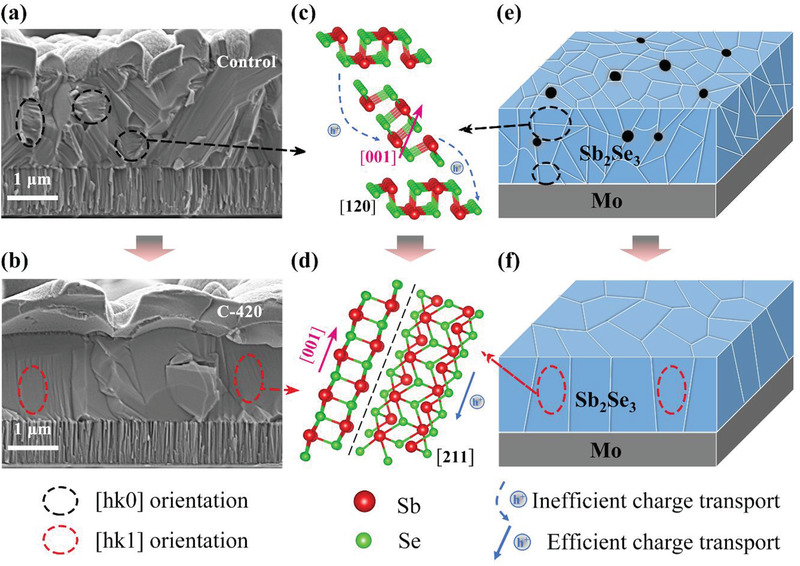
The surface/cross‐sectional morphologies and the growth orientation evolutions of the Sb_2_Se_3_ absorber layer before and after post‐selenization heat treatment. a,b) Cross‐sectional SEM image of the Control and C‐420 Sb_2_Se_3_ thin‐film devices. c,d) Atomic structures of [120]‐ and [211]‐oriented grains in Sb_2_Se_3_, charges must hop between ribbons in the [120]‐oriented grain (dashed blue traces), but are able to move smoothly along [211]‐oriented grains (solid blue traces). e,f) Schematic illustrations of the corresponding Sb_2_Se_3_ grains on Mo substrate.

Finally, the morphological and microstructural characteristics of the Mo/Sb_2_Se_3_/CdS/ITO/Ag thin‐film solar cells were investigated to further sightsee the promotion mechanism of the device performance. **Figure** **8**a,b shows the cross‐sectional SEM images of the representative Control and C‐420 Sb_2_Se_3_ devices, respectively. For Control device, Sb_2_Se_3_ light absorbing thin film consisted of multiple grains that randomly grew on Mo substrate, especially with some small grains that horizontally stacked in parallel with substrate, as marked in black circle in Figure 8a. In contrast, C‐420 Sb_2_Se_3_ thin film was composed of large and compact crystal grains that quasi‐vertically‐orientated across the whole light absorber layer, as marked in red circle in Figure 8b. Such observations matched well with the corresponding XRD results, and revealed an orientation transition from [hk0] to [hk1]. Figure 8e,f illustrate a schematic representation of the Sb_2_Se_3_ microstructure growth under post‐selenization. The VTD processed pristine thin film with grains limited, voids existed, [hk0] orientation dominated characteristics was mainly influenced by the nature of Mo substrate and deposition dynamics, i.e., growth rate. Once the post‐selenization was introduced, a certain amount of Se atoms would diffuse and react with the Se‐poor Sb_2_Se_3−_ *_x_* lattice to form stoichiometric Sb_2_Se_3_ molecules under appropriate temperature. The emerged crystals would occupy the voids first, and then start to grow into large columnar grains under proper duration. Notably, under Se atmosphere, non‐van der Waals (vdW) planes (e.g., (hkl, l≠0)) might have higher growth rates due to the increased adatoms’ absorption energy, and therefore transformed the final thin film orientation to [hk1]. For more clear comparison, two typical crystal planes of (120) and (211) were chosen to clarify the correlation between film orientation and device performance. As shown in Figure 8c,d, the [120]‐oriented grain consists of (Sb_4_Se_6_)*_n_* ribbons horizontally stacked, whereas [211]‐oriented grain consists of titled (Sb_4_Se_6_)*_n_* ribbons stacked quasi‐vertically on the substrate. Obviously, the photo‐generated carriers transport in the [211]‐oriented grains should be much easier than in the [120]‐oriented grains, since the carriers travel efficiently within the covalently bonded (Sb_4_Se_6_)*_n_* ribbons in the former condition, and a more strenuous hop between ribbons held together by vdW forces in the later scenario. Moreover, C‐420 thin film with large [211]‐oriented grains possessed fewer dangling bonds at grain boundaries, and could reduce the recombination loss.^[^ ^12^ ^]^ Overall, after an efficient post‐selenization, the optimized C‐420 Sb_2_Se_3_ thin film with large compact grains, accurate chemical composition, and benign growth orientation could be beneficial for carrier transport and recombination mitigation, corresponding to a lower *R*_s_, *N*_T_, higher *τ*, *J*_SC_, *V*_OC_, and PCE in the device.

**Figure 9:**
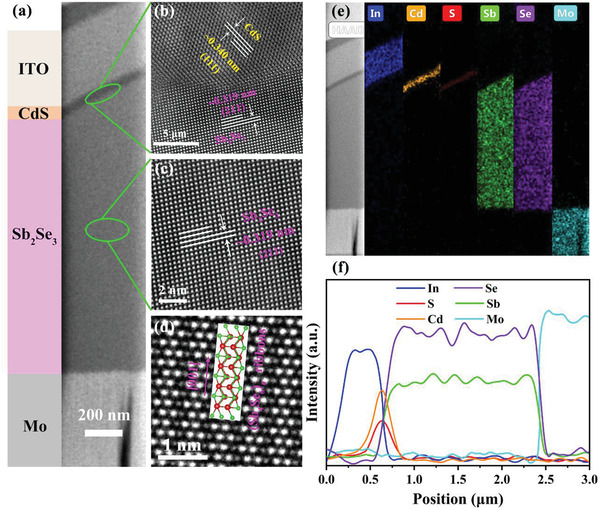
TEM characterizations of the champion C‐420 Sb_2_Se_3_ thin‐film solar cell. a) Cross‐sectional TEM image of the device. b) HRTEM image of the selected Sb_2_Se_3_/CdS heterojunction interface. c) HRTEM image, and d) HAADF‐STEM image (with Gaussian blur processed) of Sb_2_Se_3_ taken inside the bulk absorber layer region, an [001]‐oriented (Sb_4_Se_6_)*_n_* ribbon is also inset in (d). e) EDS elemental mappings and f) EDS elemental line scan profiles across the whole device, used to reveal the compositional distribution.

**Figure** **9**a shows the cross‐sectional TEM image of the champion C‐420 Sb_2_Se_3_ device, which displays an obvious layered structure with approximate thickness of 1500, 80, and 500 nm for Sb_2_Se_3_, CdS, and ITO layers, respectively. No obvious MoSe_2_ interfacial layer can be observed between Mo back contact layer and Sb_2_Se_3_ absorber layer, indicating the elimination of the common issue of high‐resistance MoSe_2_ interlayer in Mo‐based substrate structured TFPV devices.^[^ ^18^, ^21^, ^48^ ^]^ It is closely related to the two‐step thermodynamic/kinetic deposition processes, both a temperature‐dependent Mo substrate‐Sb_2_Se_3_ film interaction in pre‐VTD process, and an energetically favorable reaction between Se vapor and Sb‐rich Sb_2_Se_3_ molecule in post‐selenization process can limit the growth of MoSe_2_. Such superior back contact can also improve the carrier transport and reduce the interfacial recombination loss, echoing an acceptable *R*_s_ value of 1.79 Ω cm^−2^, and FF of 58.74%. According to the high‐resolution TEM (HRTEM) images taken at Sb_2_Se_3_/CdS interface (Figure 9b), it shows compact, well‐adherent, and pinhole‐free characteristics, which is highly beneficial for reducing carrier recombination and current leakage. The absence of lattice distortion, dislocation, or amorphous layer further validates a high‐quality Sb_2_Se_3_/CdS heterojunction. A lattice fringe with 0.340 nm interplanar d‐spacing can be assigned to (111) plane of CdS, whereas the lattice fringe with interplanar d‐spacing of 0.319 nm matches well with the (211) plane of the orthorhombic Sb_2_Se_3_. Figure 9c shows the HRTEM image corresponds to a portion at the middle region of Sb_2_Se_3_ absorber layer, quite clear lattice fringe with d‐spacing of 0.319 nm also agrees well with the (211) lattice plane, indicating an excellent continuity in the vertical direction. The Gaussian blur processed high‐angle annular dark field scanning transmission electron microscope (HAADF‐STEM) image (Figure 9d) obtained at the bulk region of Sb_2_Se_3_ clearly revealed its 1D crystal structure with (Sb_4_Se_6_)*_n_* nanoribbons stacked in parallel in the [001] direction. The “bright dots” with strong intensity can be assigned to the Sb‐dominated atomic columns (*Z*_Sb_ of 51), while the “dark dots” with much lower intensity matched well with Se‐occupied atomic columns (*Z*_Se_ of 34).^[^ ^49^ ^]^ Finally, according to TEM‐coupled EDS results (Figure 9e,f), the marked elements present reasonably compositional distribution, elements of Sb and Se also present good uniformity throughout the whole Sb_2_Se_3_ absorber layer. Overall, such morphological and microstructural observations really demonstrate the high quality of this engineered Sb_2_Se_3_ absorber layer, accompanied by excellent interface quality, leading to a remarkable improvement in device performance.

## 3 Conclusions

In summary, Sb_2_Se_3_ light absorbing thin films were successfully prepared via an effective two‐step thermodynamic/kinetic deposition process involving pre‐VTD and post‐selenization. Compared to the VTD processed Control thin film, the optimally post‐selenized C‐420 thin film showed an improved crystallinity, larger crystal grains with micro‐voids eliminated, more accurate chemical composition, more benign growth orientation of [hk1], and more suitable p‐type direct bandgap of 1.160 eV. Sb_2_Se_3_ thin‐film solar cells with substrate configuration of Mo/Sb_2_Se_3_/CdS/ITO/Ag were constructed, and the corresponding device performance were systematically investigated. Notably, an additional post‐selenization heat treatment has been found to significantly contribute to the device performance. It has demonstrated that such Sb_2_Se_3_ absorber layer growth engineering could heal the detrimental defects, simultaneously suppress the defects‐assisted interface and SCR recombination, prolong carrier lifetime and enhance charge transport. Thus, the *V*_OC_ deficit sharply decreased to a minimum value of 0.647 V, leading to a record *V*_OC_ of 0.513 V for all Sb_2_Se_3_ solar cells reported so far. Moreover, a champion device with highly interesting PCE of 7.40% is also comparable to those state‐of‐the‐art Sb_2_Se_3_ solar cells, representing the highest efficiency with VTD process in advantageous substrate structure. This interesting work can pave the way for overcoming the core bottleneck in the development of metal‐chalcogenide‐based photoelectric devices, and therefore broaden their scope of applications.

## 4 Experimental Section

### Deposition of Sb_2_Se_3_ Thin Films

Soda‐lime glass (SLG) substrates were first cleaned in an ultrasonic bath using sequential detergent, ethanol and deionized water. Mo as back contact layer was then deposited using direct current (DC) magnetron sputtering based on a dense and high‐purity Mo metallic target. A double‐pressure sputtering of Mo (≈1 µm thickness) was applied under a first 1.5 Pa pressure of 15 min to improve adhesion, and then 0.5 Pa of 25 min to ensure compactness. In subsequent, Sb_2_Se_3_ light absorber layer was deposited onto the Mo‐coated SLG substrates via VTD method. The schematic diagram of this VTD process is shown in Figure 1a, which was operated in a double‐chamber vacuum tubular furnace (Tianjin Zhonghuan Furnace Corp.). 0.5 g Sb_2_Se_3_ powder (99.999% purity, Jiangxi Ketai) was put into a quartz crucible and placed in the center of the heating zone, while the substrate was immobilized on a graphite support and located at the right end of the tube. Vacuum was pumped by a mechanical pump, and the stabilized pressure was controlled by varying the ventilation power. The sample can be obtained under a pressure of 1.3 Pa, an evaporation temperature of 540°C with ramp rate of 20°C min^−1^, a duration of 3 min, and a fixed distance (*D*) of 6 cm between substrate and source. After cooling, Sb_2_Se_3_ thin film was took out, and then underwent an additional post‐selenization heat treatment in a similar vacuum tubular furnace (Figure 1b). In detail, the VTD processed Sb_2_Se_3_ and 0.2 g of Se granule (99.999% purity, Aladdin) were separately placed into the double chambers. The chambers were evacuated using a mechanical pump, and then thoroughly purged with high‐purity argon gas. A high working pressure of 5 × 10^4^ Pa was applied to provide sufficient Se partial pressure. Temperature of the Se side was fixed at 400°C, post‐selenization of Sb_2_Se_3_ was carried out at temperatures of 380, 400, 420, and 440°C, respectively.

### Preparation of Sb_2_Se_3_ Thin‐Film Solar Cell

After the deposition of Sb_2_Se_3_ thin film, cadmium sulfide (CdS) buffer layer was deposited via chemical bath deposition (CBD) method (Figure 1c). The aqueous solution of cadmium sulfate (0.015 m), thiourea (0.75 m), and ammonium hydroxide aqueous solutions (28%) were sequentially added into deionized water, the substrates were soaked into this mixed solution and then maintained at 80°C water bath under continuous stirring for 9 min. Afterward, the specimens were rinsed with deionized water and dried in an oven. Indium tin oxide (ITO) window layer was subsequently deposited using DC magnetron sputtering under a power of 100 W, a pressure of 0.4 Pa, a flow rate of 30 sccm for argon and 8 sccm for oxygen gas, respectively. Finally, Ag electrodes were thermally evaporated onto the ITO surface to form metallic contact (Figure 1e), and the surface of the device was divided into small squares with identical active device area of 0.14 cm^2^. Overall, a schematic diagram of the device preparation process and the device configuration (Glass/Mo/Sb_2_Se_3_/CdS/ITO/Ag) is illustrated in Figure 1.

### Characterizations

The crystal structure of Sb_2_Se_3_ thin films were characterized by X‐ray diffraction (XRD, Ultima‐iv) with Cu K*_*α*_* radiation under operation conditions of 40 kV and 40 mA. Surface and cross‐sectional morphologies of the thin films were observed using scanning electron microscopy (SEM, Zeiss SUPRA 55). SEM‐coupled energy dispersive X‐ray spectroscope (EDS, BRUKER QUANTAX 200) could provide the fundamental chemical compositions. Transmission electron microscopy (TEM) characterizations were carried out on a FEI Titan Cubed Themis G2 300 microscope, wherein, the as‐measured specimen was prepared by ablating the TFPV device with focused ion beam (FIB, FEI Scios). TEM‐coupled EDS was used to further analyze the elemental distribution. Current density–voltage (*J*–*V*) characteristics of the Sb_2_Se_3_ thin‐film solar cells were measured using a multi‐meter (Keithley 2400) under AM 1.5G simulated sunlight illumination with intensity calibrated to 100 mW cm^−2^. The external quantum efficiency (EQE) spectra were acquired using a Zolix SCS101 system and a Keithley 2400 source meter. Temperature‐dependent *V*_OC_ measurements were performed on a Lakeshore 325 temperature controller, and the temperatures were swept from 340 to 160 K with a step of 20 K. An electrochemical workstation (CHI660e) was employed for the electrochemical impedance spectroscopy (EIS) measurements, which were conducted with frequency ranging from 1 Hz to 100 kHz. Capacitance–voltage (C–V) measurements were applied at an AC amplitude of 30 mV and a frequency of 10 kHz under dark condition at room temperature. The DC bias voltage during the *C*–*V* measurements was scanned from −1 to 0.3 V. Drive‐level capacitance profiling (DLCP) measurements were performed with AC amplitude from 20 to 140 mV, and a DC bias voltage from −0.2 to 0.2 V. The deep‐level transient spectroscopy (DLTS) information was obtained using an FT‐1030 HERA DLTS system configured with a JANIS VPF‐800 cryostat controller. Refection spectra of the Sb_2_Se_3_ thin films were obtained through a Shimadzu UV‐3600 UV/Vis/NIR spectrophotometer equipped with monochromator. The additional Fermi level and band level information were also analyzed through Ultraviolet photoelectron spectroscopy (UPS) using a Thermo Fisher ESCALAB 250Xi spectrometer. Transient absorption spectroscopy (TAS) characterizations were performed on a transient absorption spectrometer (Newport), which was equipped with a Spectra‐Physics Solstice Ace regenerative amplifier (800 nm wavelength, 100 fs pulses with 1 kHz repetition rate). The decay characteristics were fitted according to a biexponential model *y*  = ∑*A*_i_exp( − *x*/*t*_i_)  (*i*  =  2). Electronic structure and orbital‐resolved projected density of state (DOS) were calculated using first‐principles density functional theory (DFT) as implemented in the Vienna Ab‐initio Simulation Package (VASP).

## Conflict of Interest

The authors declare no conflict of interest.

## Supporting information

Supporting Information

## Data Availability

The data that support the findings of this study are available from the corresponding author upon reasonable request.
