# Supplementary material for: Crystal Growth Promotion and Defects Healing Enable Minimum Open‐Circuit Voltage Deficit in Antimony Selenide Solar Cells
Source: Adv Sci (Weinh). 2022 Jan 28;9(9):2105142. doi: 10.1002/advs.202105142 (PMC8948594; doi:10.1002/advs.202105142)
Supplement: Supplementary file 1 — Supporting Information [file ADVS-9-2105142-s001.pdf]

ADVANCED SCIENCE
Open Access

Supporting Information

for *Adv. Sci.*, DOI 10.1002/advs.202105142

Crystal Growth Promotion and Defects Healing Enable Minimum Open-Circuit Voltage Deficit in Antimony Selenide Solar Cells

*Guangxing Liang, Mingdong Chen, Muhammad Ishaq, Xinru Li, Rong Tang, Zhuanghao Zheng, Zhenghua Su, Ping Fan, Xianghua Zhang and Shuo Chen**

ADVANCED SCIENCE
Open Access

Supporting Information

for *Adv. Sci.*, DOI: 10.1002/advs.202105142

Crystal Growth Promotion and Defects Healing Enable Minimum Open-Circuit Voltage Deficit in Antimony Selenide Solar Cells

*Guangxing Liang, Mingdong Chen, Muhammad Ishaq, Xinru Li, Rong Tang, Zhuanghao Zheng, Zhenghua Su, Ping Fan, Xianghua Zhang and Shuo Chen**

Copyright WILEY-VCH Verlag GmbH & Co. KGaA, 69469 Weinheim, Germany, 2018.

Supporting Information

Crystal Growth Promotion and Defects Healing Enable Minimum Open-Circuit Voltage Deficit in Antimony Selenide Solar Cells

*Guangxing Liang, Mingdong Chen, Muhammad Ishaq, Xinru Li, Rong Tang, Zhuanghao Zheng, Zhenghua Su, Ping Fan, Xianghua Zhang and Shuo Chen**

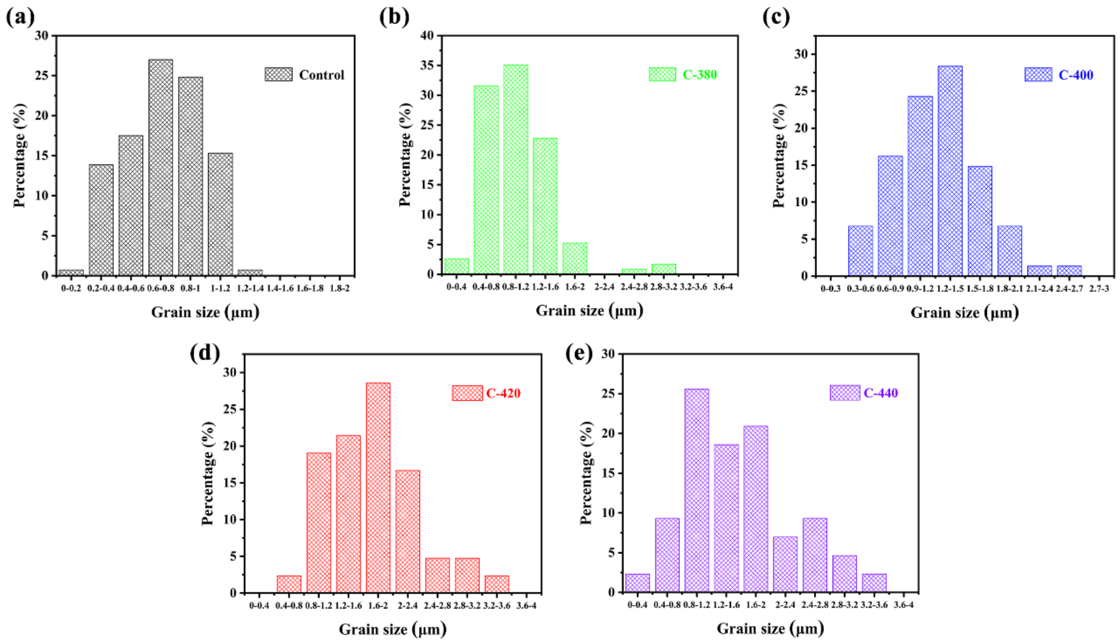

Figure S1. Grain size distributions of the Sb_2Se_3 thin films with different post-selenization temperatures.

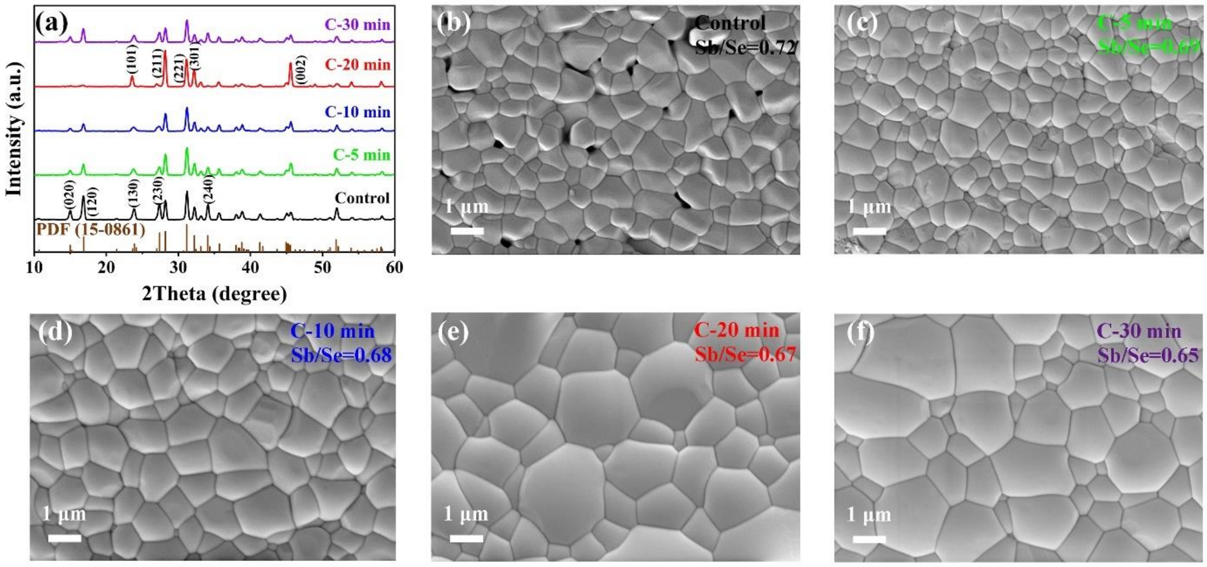

Figure S2. (a) XRD patterns, (b-f) top-view SEM images of the Sb_2Se_3 thin films with different post-selenization durations, labeled as Control (0 min), C-5 min, C-10 min, C-20 min and C-30 min, respectively.

Supplementary note 1. DFT calculations

Density functional theory (DFT) calculations were performed using the Vienna Ab-initio Simulation Package (VASP) software.[1,2] The exchange correlation functional was approximated by using the generalized gradient approximation (GGA) with Perdew-Burke-Ernzerhof (PBE) parametrization. The cutoff energy of the plane-wave was set to be 500 eV to ensure convergence. The convergence criteria of the force and the energy were 10^{-3} eV/Å and 10^{-6} eV, respectively. The 3D Brillouin zone integration was done with a k-mesh density of 100/a, where a denoted the length of the lattice constant in the unit of Å. The Sb-rich Sb_2Se_3 was modeled from Sb_8Se_{11} with a Se-vacancy in Sb_2Se_3 unit cell (Figure S3a), which was close to the experiment determined Sb/Se ratio of 0.72, and the lattice parameters were a = 13.36 Å, b = 4.00 Å and c = 11.41 Å. Meanwhile, as shown in Figure S3b, the stoichiometric Sb_2Se_3 was modeled from Sb_8Se_{12} with optimized lattice parameters of a = 12.84 Å, b = 4.03 Å and c = 11.54 Å for the unit cell.

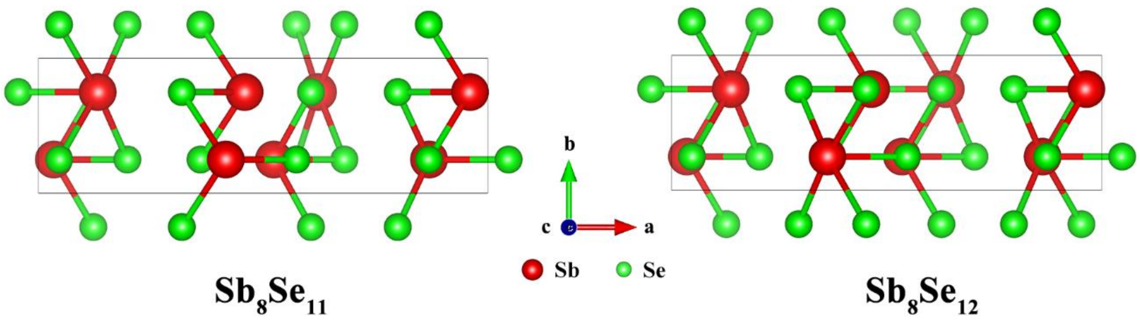

Figure S3. The atomic configurations of (a) Sb_8Se_{11}, and (b) Sb_8Se_{12}, used in DFT calculations.

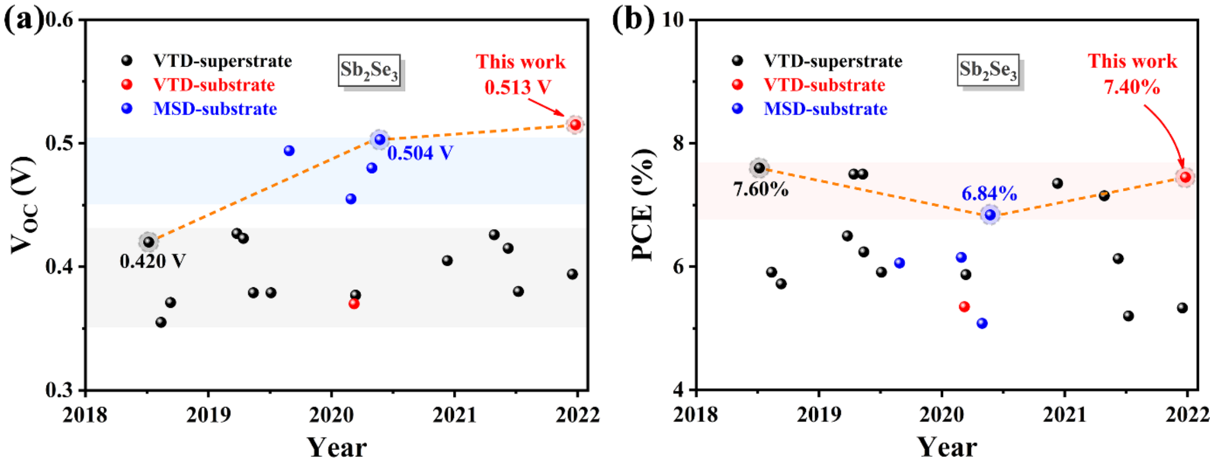

Figure S4. (a) V_{OC}, and (b) PCE statistics of the Sb_2Se_3 solar cells prepared by representative VTD and magnetron sputtering deposition (MSD) methods.[3-20] The dashed circle marked black, red, and blue points represent the champion PCE and corresponding V_{OC} of the VTD processed superstrate, VTD processed substrate, and MSD processed substrate Sb_2Se_3 solar cells, respectively.

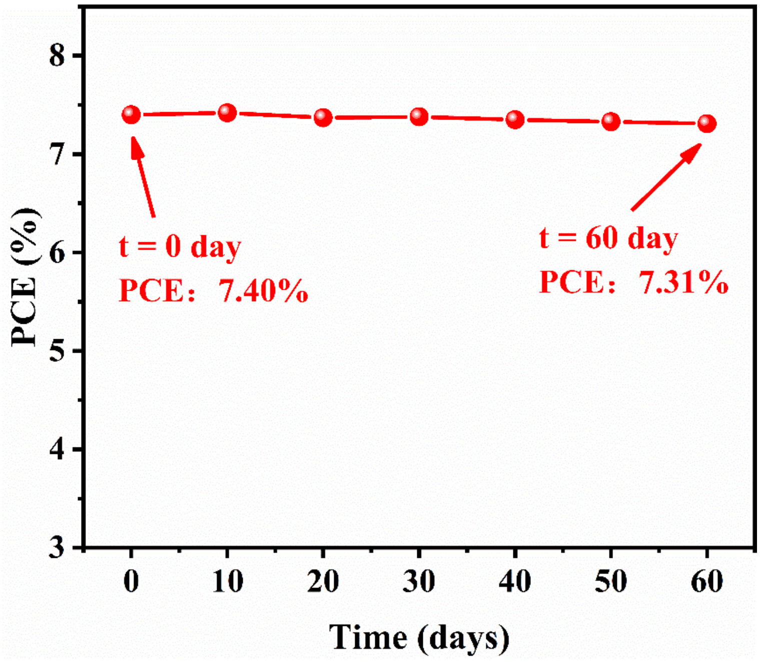

Figure S5. PCE evolution of the C-420 device after 60 days storage in ambient air without special encapsulation.

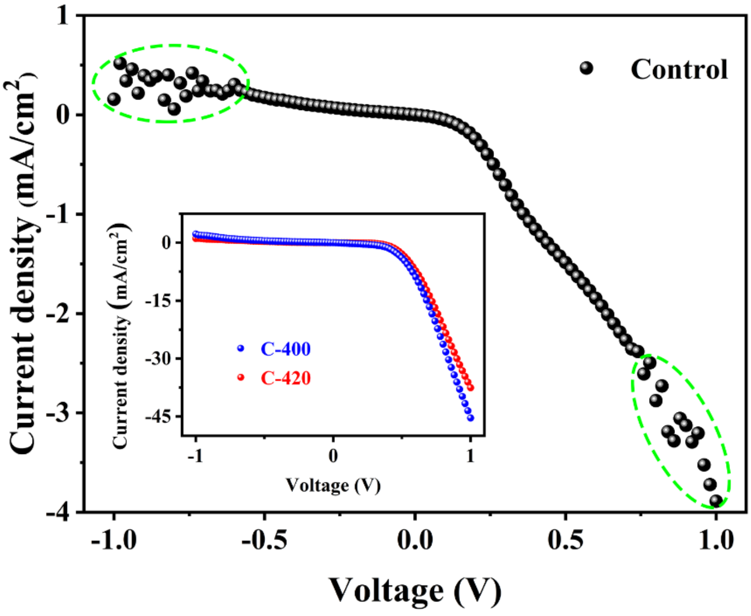

Figure S6. *J-V* curve of the Control device measured in dark condition, covering voltage range from −1 V to 1 V. *J-V* curves of the C-400 and C-420 devices obtained at the same condition are inset in the upper right corner for comparison.

Supplementary note 2. Ultraviolet photoelectron spectroscopy

Ultraviolet photoelectron spectroscopy (UPS) was carried out to investigate the band structure of Control (Sb-rich) and C-420 (Se-rich) Sb_2Se_3 thin films, including the conduction band (E_C), valence band (E_V) and Fermi level (E_F). According to the secondary electron cut-off (SEC) edge and valence band (VB) position (Figure S7a and b), the E_F were determined as −4.41 and −4.66 eV for Control Sb_2Se_3 and C-420 Sb_2Se_3, respectively. Combined with their optical bandgap (E_g) values, E_C and E_V of the Control sample were calculated as −3.98 and −5.12 eV, whereas showed a slight up-shift to −3.92 and −5.08 eV for C-420 sample. Thus, an obvious transition of the conductive type from n-type to p-type can be observed, which was closely related to the chemical composition evolution after post-selenization heat treatment of the Sb_2Se_3 thin film.

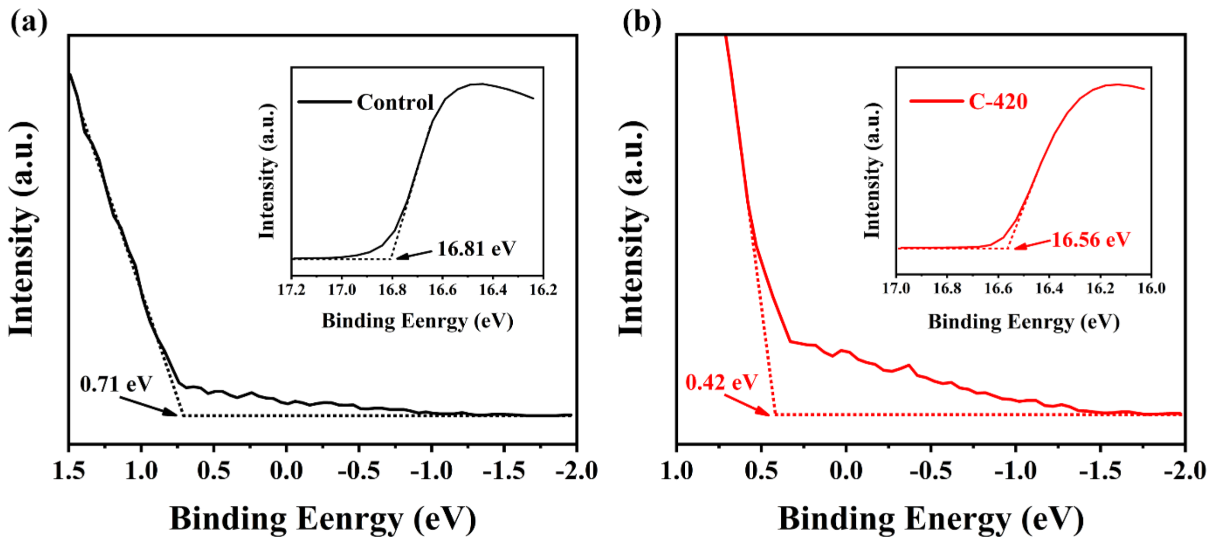

Figure S7. UPS characterizations: secondary electron cut-off (SEC) edge and valence band (VB) position of the Control (a), and C-420 (b) Sb_2Se_3 thin films.

Supplementary note 3. Background of DLTS measurements

Herein, deep-level transient spectroscopy (DLTS) was conducted to analyze the deep defects in the Sb_2Se_3 absorber layer of Control and C-420 devices. Arrhenius plots obtained from defect peaks in DLTS signal are shown in Figure 7b. The active energy (E_A, E_C–E_T or E_T–E_V) and capture cross section (σ) of electron and hole defects can be calculated from the Arrhenius plots according to the following equations[21]

$$\ln(\tau_e v_{th,n} N_C) = \frac{E_C - E_T}{k_B T} - \ln(X_n \sigma_n) \tag{1}$$

$$\ln(\tau_e v_{th,p} N_V) = \frac{E_T - E_V}{k_B T} - \ln(X_p \sigma_p) \tag{2}$$

where τ_e, N_C, N_V, E_C, E_V and E_T are emission time constant, conduction band state density, valence band state density, conduction band, valence band, and trap energy level, respectively. X_n and X_p are the entropy factor of hole and electron, σ_n and σ_p represent the capture cross-section of electron and hole defects, respectively. T and k_B are the temperature and Boltzmann constant, respectively. $v_{th,n}$ and $v_{th,p}$ are the thermal velocities associated with electron and hole defects. The $v_{th,n}$ and N_C can be obtained by following equations

$$v_{th,n} = \sqrt{\frac{3k\mathrm{T}}{m_n{}^*}} \tag{3}$$

$$N_C = 2\left(\frac{2\pi m_n{}^* kT}{h^2}\right)^{\frac{3}{2}} \tag{4}$$

where $m_n{}^*$ is the effective mass for electrons, with similar equations for $v_{th,p}$ and N_V. The activation energy of electron (E_C–E_T) and hole (E_T–E_C) traps were obtained from the slopes of equations (2) and (3) through linear regression. σ_n and σ_p values can be extracted from the intersection with y-axis. Defect concentration (N_T) can be obtained from following equations[22]

$$N_T = 2N_S \frac{\Delta C}{C_R} \tag{5}$$

$$N_S = \frac{2C^2}{q\varepsilon A^2}(V + V_d) \tag{6}$$

where N_T and N_S represents the trap density and the shallow dopant concentration,

respectively. C_R is the capacitance under reverse bias, and ΔC is the amplitude of capacitance transient. V and V_d are the applied voltage and diffusion voltage, respectively.

Table S1. Fitted parameters for transient kinetic traces of the Control and C-420. The average lifetime τ was calcultated according to the equation: $\tau = (A_1\tau_1^2 + A_2\tau_2^2)/(A_1\tau_1 + A_2\tau_2)$.

**Sample**	A_1	τ_1 **(ps)**	A_2	τ_2 **(ps)**	τ **(ps)**
Control	0.79	349	0.21	3548	2684
C-420	0.53	487	0.47	15010	14497

Table S2. A comparison of TAS measured carrier lifetime (τ) of antimony chalcogenides.

**Materials**	**Preparation**	**Substrate**	τ **(ps)**	**Method**	**Ref.**
Sb_2S_3	Solution	Glass	23000	TAS	[23]
	Thermal evaporation	FTO	18700	TAS	[24]
Sb_2Se_3	VTD	CdS	1339	TAS	[3]
	Solution	FTO	6235	TAS	[25]
	VTD-Se	Glass	14497	TAS	This work
$Sb_2(S,Se)_3$	Hydrothermal	FTO	10604	TAS	[21]
	Hydrothermal	FTO	9546	TAS	[26]

References

[1] A. Stoliaroff, A. Lecomte, O. Rubel, S. Jobic, X. Zhang, C. Latouche, X. Rocquefelte, *ACS Appl. Energy Mater*. **2020**, *3*, 2496.

[2] Z. Cai, C.-M. Dai, S. Chen, *Sol. RRL* **2020**, *4*, 1900503.

[3] X. Wen, C. Chen, S. Lu, K. Li, R. Kondrotas, Y. Zhao, W. Chen, L. Gao, C. Wang, J. Zhang, G. Niu, J. Tang, *Nat. Commun.* **2018**, *9*, 2179.

[4] J. Zhang, R. Kondrotas, S. Lu, C. Wang, C. Chen, J. Tang, *Sol. Energy* **2019**, *182*, 96.

[5] K. Li, S. Wang, C. Chen, R. Kondrotas, M. Hu, S. Lu, C. Wang, W. Chen, J. Tang, *J. Mater. Chem. A* **2019**, *7*, 9665.

[6] H. Guo, X. Jia, S. H. Hadke, J. Zhang, W. Wang, C. Ma, J. Qiu, N. Yuan, L. H. Wong, J. Ding, *J. Mater. Chem. C* **2020**, *8*, 17194.

[7] X. Wen, Z. Lu, G.-C. Wang, M. A. Washington, T.-M. Lu, *Nano Energy* **2021**, *85*, 106019.

[8] X. Hu, J. Tao, Y. Wang, J. Xue, G. Weng, C. Zhang, S. Chen, Z. Zhu, J. Chu, *Appl. Mater. Today* **2019**, *16*, 367.

[9] K. Li, F. Li, C. Chen, P. Jiang, S. Lu, S. Wang, Y. Lu, G. Tu, J. Guo, L. Shui, Z. Liu, B. Song, J. Tang, *Nano Energy* **2021**, *86*, 106101.

[10] R. Tang, Z.-H. Zheng, Z.-H. Su, X.-J. Li, Y.-D. Wei, X.-H. Zhang, Y.-Q. Fu, J.-T. Luo, P. Fan, G.-X. Liang, *Nano Energy* **2019**, *64*, 103929.

[11] Y.-D. Luo, R. Tang, S. Chen, J.-G. Hu, Y.-K. Liu, Y.-F. Li, X.-S. Liu, Z.-H. Zheng, Z.-H. Su, X.-F. Ma, P. Fan, X.-H. Zhang, H.-L. Ma, Z.-G. Chen, G.-X. Liang, *Chem. Eng. J.* **2020**, *393*, 124599.

[12] G.-X. Liang, Y.-D. Luo, S. Chen, R. Tang, Z.-H. Zheng, X.-J. Li, X.-S. Liu, Y.-K. Liu, Y.-F. Li, X.-Y. Chen, Z.-H. Su, X.-H. Zhang, H.-L. Ma, P. Fan, *Nano Energy* **2020**, *73*, 104806.

[13] W. Wang, Z. Cao, H. Wang, J. Luo, Y. Zhang, *J. Mater. Chem. A* **2021**, *9*, 26963.

[14] X. Hu, J. Tao, S. Chen, J. Xue, G. Weng, Kaijiang, Z. Hu, J. Jiang, S. Chen, Z. Zhu, J. Chu, *Sol. Energy Mater. Sol. Cells* **2018**, *187*, 170.

[15] J. Tao, X. Hu, J. Xue, Y. Wang, G. Weng, S. Chen, Z. Zhu, J. Chu, *Sol. Energy Mater. Sol. Cells* **2019**, *197*, 1.

[16] C. Wang, S. Lu, S. Li, S. Wang, X. Lin, J. Zhang, R. Kondrotas, K. Li, C. Chen, J. Tang, *Nano Energy* **2020**, *71*, 104577.

[17] J. Kim, S. Ji, Y. Jang, G. Jeong, J. Choi, D. Kim, S.-W. Nam, B. Shin, *Sol. RRL* **2021**, *5*, 2100327.

[18] J. Tao, X. Hu, Y. Guo, J. Hong, K. Li, J. Jiang, S. Chen, C. Jing, F. Yue, P. Yang, C. Zhang, Z. Wu, J. Tang, J. Chu, *Nano Energy* **2019**, *60*, 802.

[19] Q. Cang, H. Guo, X. Jia, H. Ning, C. Ma, J. Zhang, N. Yuan, J. Ding, *Sol. Energy* **2020**, *199*, 19.

[20] G. Liang, X. Chen, R. Tang, Y. Liu, Y. Li, P. Luo, Z. Su, X. Zhang, P. Fan, S. Chen, *Sol. Energy Mater. Sol. Cells* **2020**, *211*, 110530.

[21] R. Tang, X. Wang, W. Lian, J. Huang, Q. Wei, M. Huang, Y. Yin, C. Jiang, S. Yang, G. Xing, S. Chen, C. Zhu, X. Hao, M. A. Green, T. Chen, *Nat. Energy* **2020**, *5*, 587.

[22] Y. Zhao, S. Yuan, D. Kou, Z. Zhou, X. Wang, H. Xiao, Y. Deng, C. Cui, Q. Chang, S. Wu, *ACS Appl. Mater. Interfaces* **2020**, *12*, 12717.

[23] Z. Yang, X. Wang, Y. Chen, Z. Zheng, Z. Chen, W. Xu, W. Liu, Y. Yang, J. Zhao, T. Chen, H. Zhu, *Nat. Commun.* **2019**, *10*, 4540.

[24] W. Lian, C. Jiang, Y. Yin, R. Tang, G. Li, L. Zhang, B. Che, T. Chen, *Nat. Commun.* **2021**, *12*, 3260.

[25] Y. Ma, B. Tang, W. Lian, C. Wu, X. Wang, H. Ju, C. Zhu, F. Fan, T. Chen, *J. Mater. Chem. A* **2020**, *8*, 6510.
[26] Y. Zhao, S. Wang, C. Jiang, C. Li, P. Xiao, R. Tang, J. Gong, G. Chen, T. Chen, J. Li, X. Xiao, *Adv. Energy Mater.* **2021**, 2103015.
